# Impact of prehabilitation during neoadjuvant chemotherapy and interval cytoreductive surgery on ovarian cancer patients: a pilot study

**DOI:** 10.1186/s12957-022-02517-1

**Published:** 2022-02-23

**Authors:** Ester Miralpeix, Josep-Maria Sole-Sedeno, Cristina Rodriguez-Cosmen, Alvaro Taus, Maria-Dolors Muns, Berta Fabregó, Gemma Mancebo

**Affiliations:** 1 Department of Obstetrics and Gynecology, Hospital del Mar, Universitat Autònoma de Barcelona, Passeig Marítim 25-29, E-08003 Barcelona, Spain; 2 Universitat Pompeu Fabra, Barcelona, Spain; 3 Department of Anesthesia, Hospital del Mar, Barcelona, Spain; 4 Department of Medical Oncology, Hospital del Mar, Barcelona, Spain; 5 Cancer Research Program, IMIM (Hospital del Mar Institute of Medical Research), Barcelona, Spain; 6 Department of Endocrinology, Hospital del Mar, Barcelona, Spain

**Keywords:** Prehabilitation, Neoadjuvant chemotherapy, Interval cytoreductive surgery, Ovarian cancer

## Abstract

**Background:**

Cytoreductive surgery followed by systemic chemotherapy is the standard of treatment in advanced ovarian cancer where feasible. Neoadjuvant chemotherapy (NACT) followed by surgery is applicable where upfront cytoreductive surgery is not feasible because of few certain reasons. Nevertheless, surgical interventions and the chemotherapy itself may be associated with postoperative complications usually entailing slow postoperative recovery. Prehabilitation programs consist of the patient’s preparation before surgery to improve the patient’s functional capacity. The aim of this study was to evaluate the impact of a prehabilitation program during neoadjuvant treatment and interval cytoreductive surgery for ovarian cancer patients.

**Methods:**

A retrospective observational pilot study of patients with advanced ovarian cancer treated with NACT and interval cytoreductive surgery was conducted. The prehabilitation group received a structured intervention based on physical exercise, nutritional counseling, and psychological support. Nutritional parameters were assessed preoperatively and postoperatively, and functional parameters and perioperative and postoperative complications were also recorded.

**Results:**

A total of 29 patients were included in the study: 14 in the prehabilitation group and 15 in the control group. The patients in the prehabilitation program showed higher mean total protein levels in both preoperative (7.4 vs. 6.8, *p* = 0.004) and postoperative (4.9 vs. 4.3, *p* = 0.005) assessments. Up to 40% of controls showed intraoperative complications vs. 14.3% of patients in the prehabilitation group, and the requirement of intraoperative blood transfusion was significantly lower in the prehabilitation group (14.3% vs. 53.3%, *p* = 0.027). The day of the first ambulation, rate of postoperative complications, and length of hospital stay were similar between the groups. Finally, trends towards shorter time between diagnosis and interval cytoreductive surgery (*p* = 0.097) and earlier postoperative diet restart (*p* = 0.169) were observed in the prehabilitation group.

**Conclusion:**

Prehabilitation during NACT in women with ovarian cancer candidates to interval cytoreductive surgery may improve nutritional parameters and thereby increase postoperative recovery. Nevertheless, the results of this pilot study are preliminary, and further studies are needed to determine the clinical impact of prehabilitation programs.

## Introduction

Advanced ovarian cancer is a complex and challenging disease whose treatment requires a multimodal approach [1]. Primary treatment consists of optimal primary cytoreductive surgery followed by systemic chemotherapy. In patients unsuitable for primary cytoreductive surgery due to advanced age, frailty, poor performance status, comorbidities, or disease that is unlikely to be optimally cytoreduced, neoadjuvant chemotherapy (NACT) followed by interval cytoreductive surgery should be considered [2, 3]. Therefore, cytoreductive surgery after NACT is often undertaken in patients who are physically, nutritionally, and/or psychologically affected. The aim of NACT is to achieve radiological and clinical improvement to increase the likelihood of optimal cytoreduction at interval cytoreductive surgery [4, 5].

Many patients with advanced ovarian cancer present with abdominal disease that can lead to malnutrition, and some patients present physiological effects of peripheral muscle wasting, decreased exercise tolerance and fatigue, and psychological morbidity for anxiety of a potentially terminal cancer diagnosis [6]. Surgery is an aggressive procedure that disrupts the physiologic status and triggers a general stress response, altering hormonal, metabolic, immunologic, and neurological functions, and NACT treatment may also affect the physiologic status and body function [7]. In particular, cytoreductive surgery usually requires peritonectomy, lymphadenectomy, visceral resection, and gastrointestinal anastomoses [8, 9]. Therefore, it seems appropriate to restore the baseline status of ovarian cancer patients before exposition to another acute stressor such as surgery and especially after NACT. The period on NACT before interval cytoreductive surgery offers a window of opportunity to improve the patient’s functional capacity or to restore its capacity to baseline levels [10, 11].

Prehabilitation programs consist of the patient’s preparation between diagnosis and surgery to improve functional capacity and metabolic reserves before the intervention, resulting in a reduction of perioperative complications. Multimodal prehabilitation may include exercise, nutritional counseling, psychological support, and optimization of underlying conditions, as well as cessation of negative health behaviors such as alcohol or tobacco consumption [12]. Emerging evidence suggests that multimodal prehabilitation programs in major cancer surgeries have a positive impact on the patients’ outcomes [13, 14]. Therefore, it would be expected that a multimodal prehabilitation program during NACT before interval cytoreductive surgery in patients with advanced ovarian cancer could enhance patients’ functional capacity. With this purpose, we formed a multidisciplinary team to implement a pilot prehabilitation program to support gynecologic oncology patients. The aims of this study were to evaluate our initial experience and impact of a multimodal prehabilitation program in advanced-stage ovarian cancer patients undergoing interval cytoreductive surgery.

## Materials and methods

### Design and subjects

A retrospective pilot observational study of patients undergoing interval cytoreductive surgery for ovarian cancer was conducted at the Hospital del Mar Barcelona between January 2015 and June 2020.

Eligible patients were women diagnosed with advanced ovarian cancer undergoing interval cytoreductive surgery after standardized 3 cycles of NACT with carboplatin and paclitaxel. In previous interval cytoreductive surgery, all patients were evaluated through laparoscopic approach and computed tomography scan to consider optimally resectable disease by a multidisciplinary team. The exclusion criteria were inability to give informed consent, having non-resectable disease, inability to perform exercises with locomotor limitations, cognitive deterioration impeding adherence to the program, patients who declined surgery or NACT, or those who received non-standard NACT. The prehabilitation program was implemented in January 2018, and the first patients that followed the prehabilitation program were consecutively included in this pilot study. Patients treated before this date were used as a control group.

### Prehabilitation intervention

The prehabilitation program was developed by a multidisciplinary team of gynecologists, anesthesiologists, physiotherapists, dieticians, psychologists, and geriatricians. The prehabilitation group received a structured intervention including physical exercise recommendations, nutritional counseling, and psychological support. This program was extensively described in a previous publication [15]. Briefly, all patients included in the prehabilitation program received recommendations for daily exercise practice, nutritional counseling based on homemade recipes of protein supplementation and psychological support, and a preoperative carbohydrate loading and an inspiratory threshold-loading device in our consultation. Patients in the prehabilitation program received perioperative care, following the guidelines of the Enhanced Recovery After Surgery (ERAS) program, which has been the standard of surgical approach for our patients since 2015 [16, 17]. The control group received standard of care, and no specific intervention before surgery was administered.

The length of the prehabilitation program was not fixed and depended on the patient’s status, tumor type, and extension. In addition, the duration also depended on the time period before surgery, which was modulated by NACT tolerance and toxicity, organizational aspects of healthcare providers, and by type of surgical intervention.

### Variables and outcomes

Demographic and clinical baseline information was collected retrospectively from medical registries. Nutritional status was evaluated by total serum protein, albumin, hemoglobin, and prealbumin levels. Measurements were recorded at diagnosis, just before the interval surgery, 48–72 h post-surgery, and 1 month after surgery. The main surgical, intraoperative, and postoperative parameters reported included peri- and postoperative complications, postoperative pain, length of hospital stay, days of intensive care unit (ICU) stay, day of the first ambulation, and readmission rates. Complications were classified according to Clavien-Dindo classification (grades I–V) [18]. The complexity of the surgical procedure was classified using Aletti’s surgical complexity score (SCS), based on the number and the complexity of the surgical procedures performed [19].

### Statistical methods

Statistical analysis was performed using SPSS 21.0 (Chicago, IL, USA) assuming a statistically significant level of 5% (*p* < 0.05). Participant demographic and clinical characteristics were summarized using descriptive statistics. Continuous variables were reported as mean (range) or mean ± standard deviation (SD) when indicated, and categorical variables were reported as frequency and percentage (%). Pearson’s chi-square test or Fisher exact test was used to compare categorical variables, and Student’s *t*-test or non-parametric Mann-Whitney test was used for continuous variables, when appropriate.

### Ethical considerations

The study was evaluated and approved by the institutional ethics committee (Institutional Review Board Project No. 2017/7770/I). All participants provided a written informed consent.

## Results

A total of 29 patients undergoing interval cytoreductive surgery for ovarian cancer were included in the study. Overall, 15 patients were included in the control group and 14 in the prehabilitation group. The mean age of patients was 64.5 ± 8.8 years (range 51–83). Baseline demographic and clinical characteristics of the study population are shown in Table 1. There were no significant differences in age, clinical disease stage, or comorbidities between the groups. It should be mentioned that the mean time between diagnosis and interval cytoreductive surgery was shorter in the prehabilitation group than in controls (13.5 vs. 15.3 weeks, *p* = 0.097). During this time period, patients were on NACT treatment, and the intervention group followed the prehabilitation recommendations. Thus, control patients required more time on NACT before the surgery was performed, although it was not statistically significant.

**Table 1:** Baseline demographic and clinical characteristics of the study population

	Control group (*n* = 15)	Prehabilitation group (*n* =14)	***p-***value
Age (years), mean ± SD	63.8 ± 7.6	65.1 ± 10.2	0.689
BMI (kg/m^2^), mean ± SD	27.2 ± 5.7	29.3 ± 6.1	0.360
Disease stage, *n* (%)			
III	8 (53.3)	7 (50.0)	0.858
IV	7 (46.7)	7 (50.0)	
Medical history, *n* (%)			
Heart disease	2 (13.3)	1 (7.1)	0.584
Diabetes	2 (13.3)	3 (21.4)	0.564
Anticoagulation	2 (13.3)	0 (0)	0.157
Smoking	6 (40.0)	3 (21.4)	0.270
Alcohol	2 (13.3)	0 (0)	0.157
ASA class, *n* (%)			
II	6 (40.0)	9 (64.3)	0.323
III	8 (53.3)	5 (35.7)	
IV	1 (6.7)	0 (0)	
Time between diagnosis and interval surgery (weeks), mean ± SD [range]	15.3 ± 3.2 [11.3–24.3]	13.5 ± 2.0 [11.4–18.0]	0.097

*ASA* American Society of Anesthesiologists, *BMI* body mass index, *COPD* chronic obstructive pulmonary disease, *SD* standard deviation

The results comparing the impact of the prehabilitation program on nutritional parameters are shown in Table 2. Prehabilitation group patients received a preoperative carbohydrate loading; however, this was not associated with an increased intraoperative risk of hyperglycemia (*p* = 0.668), nor postoperative insulin requirement (*p* = 0.782). In addition, the mean value of preoperative Hb1Ac was not different between the groups (*p* = 0.382), and no significant differences in serum total proteins, albumin, prealbumin, and hemoglobin levels were noted at diagnosis.

**Table 2:** Impact of the prehabilitation program on nutritional parameters

		Control group (*n* = 15)	Prehabilitation group (*n* = 14)	***p-***value
Preoperative HbA1c (%)		5.6 ± 0.6	5.8 ± 0.6	0.382
Intraoperative glucose (BMTest) (mg/dl)		162.7 ± 38.0	154.7 ± 53.5	0.668
Postoperative insulin requirement, *n* (%)		5 (33.3%)	4 (28.6%)	0.782
**Diagnosis**	Total protein (g/dl)	6.7 ± 0.7	6.8 ± 0.4	0.510
	Albumin (g/dl)	3.9 ± 0.5	3.9 ± 0.4	0.959
	Prealbumin (mg/dl)	15.6 ± 6.2	14.2 ± 6.6	0.683
	Hemoglobin (g/dl)	12.0 ± 1.8	12.2 ± 1.6	0.843
**Preoperative**	Total protein (g/dl)	6.8 ± 0.5	7.4 ± 0.3	0.004
	Albumin (g/dl)	4.1 ± 0.5	4.4 ± 0.3	0.209
	Prealbumin (mg/dl)	20.2 ± 4.9	22.4 ± 4.3	0.428
	Hemoglobin (g/dl)	10.8 ± 1.0	10.8 ± 0.9	0.915
**Postoperative**	Total protein (g/dl)	4.3 ± 0.7	4.9 ± 0.6	0.005
	Albumin (g/dl)	2.4 ± 0.6	2.8 ± 0.4	0.021
	Prealbumin (mg/dl)	9.5 ± 3.3	12.5 ± 4.3	0.124
	Hemoglobin (g/dl)	9.5 ± 1.0	8.9 ± 1.3	0.164
**One month postoperative**	Total protein (g/dl)	7.0 ± 0.7	7.2 ± 0.5	0.693
	Albumin (g/dl)	4.2 ± 0.5	4.2 ± 0.4	0.985
	Prealbumin (mg/dl)	20.7 ± 8.2	22.2 ± 5.3	0.465
	Hemoglobin (g/dl)	11.2 ± 1.0	11.2 ± 1.4	0.683

Data are presented as mean ± SD, unless otherwise specified

**Fig. 1:**
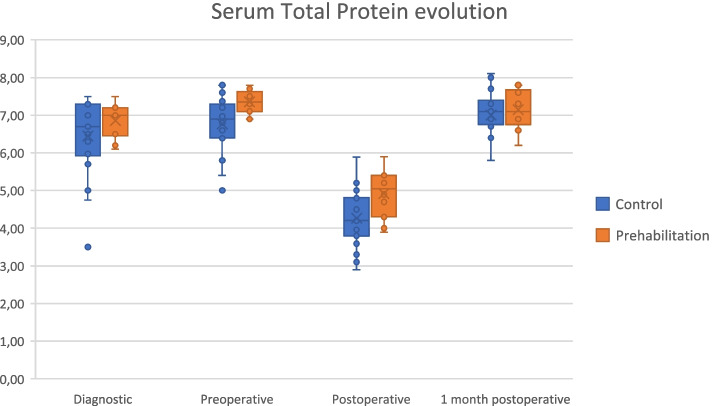
Serum total protein value evolution

In contrast, the prehabilitation group showed higher preoperatively protein levels compared to the control group (7.4 vs. 6.8, *p* = 0.004). In addition, postoperative serum total proteins and albumin values were higher in the prehabilitation group (*p* = 0.005 and *p* = 0.021, respectively). Although not significant, a trend to higher postoperative levels of prealbumin and hemoglobin was also detected in the prehabilitation group. Figure 1 shows the total serum protein value evolution in the control and prehabilitation groups.

Regarding the impact of the prehabilitation program on surgical and intraoperative parameters, no significant differences in length of surgery or surgical procedure complexity between the groups were observed (Table 3). However, although preoperative hemoglobin levels were similar between the groups, the prehabilitation group showed a significantly lower intraoperative blood transfusion rate (14.3%) compared with the control group (53.3%) (*p* = 0.027), as well as a trend towards lower intraoperative vasoactive drug requirement (7.1% vs. 20.0%). The incidence of intraoperative complications showed a trend towards fewer complications in the prehabilitation group (40% of patients in the control group vs. 14.3% in the prehabilitation group).

**Table 3:** Intraoperative and surgical outcomes

	Control group (*n* = 15)	Prehabilitation group (*n* = 14)	***p-***value
**Surgical approach**			
Laparoscopic	2 (13.3)	0 (0)	0.157
Laparotomic	13 (86.7)	14 (100)	
**Intestinal resection**	1 (6.7)	2 (14.3)	0.501
**Epidural anesthesia**	11 (73.3)	11 (78.6)	0.742
**Surgical time** (min) mean ± SD	309.0 ± 76.7	300.0 ± 87.7	0.770
**Aletti’s SCS**, mean ± SD	5.0 ± 1.2	5.1 ± 2.6	0.927
**Intraoperative blood transfusion**	8 (53.3)	2 (14.3)	0.027
**Intraoperative vasoactive drugs**	3 (20.0)	1 (7.1)	0.316
**Intraoperative complications**	6 (40.0)	2 (14.3)	0.122
Vascular	3 (20.0)	1 (7.1)	0.316
Intestinal	2 (13.3)	1 (7.1)	0.584
Urological	1 (6.7)	1 (7.1)	0.960
Cardiovascular	0 (0)	0 (0)	-

Data are presented as *n* (%), unless otherwise specified

*SCS* surgical complexity score, *SD* standard deviation

When considering the postoperative outcomes, no significant differences regarding the length of stay, time to start adjuvant chemotherapy, and postoperative pain control between the groups were observed. Additionally, prehabilitation was not associated with increased risk of postoperative complications, reintervention, readmission rate, or mortality, although one patient in the control group died due to severe postoperative complications and none in the prehabilitation group. To note, the incidence of postoperative complications was similar between the groups, according to the Clavien-Dindo classification.

Furthermore, the prehabilitation group showed a trend towards earlier postoperative diet restart compared with the control group (1.3 days vs. 1.7 days). All postoperative and functional results are shown in Table 4.

**Table 4:** Postoperative outcomes

	Control group, mean ± SD (*n* = 15)	Prehabilitation group, mean ± SD (*n* = 14)	***p-***value
**Hospital stay** (days)	7.8 ± 6.8	7.4 ± 5.0	0.700
**ICU stay** (days)	1.2 ± 0.6	1.2 ± 0.9	0.959
**Time to start CT of surgery day** (days)	39.9 ± 14.3	37.4 ± 9.2	0.602
**Pain VAS day 1**	1.6 ± 1.7	1.7 ± 1.7	0.856
**Pain VAS day 2**	1.2 ± 1.0	1.4 ± 1.4	0.736
**Diet restart** (days)	1.7 ± 0.8	1.3 ± 0.6	0.169
**Deambulation restart** (days)	3.1 ± 1.2	2.9 ± 1.4	0.678
**Postoperative complications,** ***n*** **(%)**			
Non reported	6 (40.0)	4 (28.6)	0.518
Reported	9 (60.0)	10 (71.4)	
**Postoperative complications**			
Paralytic ileus	2 (13.3)	1 (7.1)	0.584
Surgical site infection (superficial and deep)	6 (40.0)	5 (35.7)	0.812
Surgical site infection (organ and space)	2 (13.3)	0 (0)	0.157
Anastomosis breakdown	2 (13.3)	0 (0)	0.157
Cardiovascular	1 (6.7)	0 (0)	0.326
Respiratory	3 (20.0)	1 (7.1)	0.316
Neurological	0 (0)	2 (14.3)	0.129
Urinary tract infection	4 (26.7)	1 (7.1)	0.164
Multiorgan failure	1 (6.7)	0 (0)	0.326
**Surgical reintervention**	1 (6.7)	1 (7.1)	0.960
**Clavien-Dindo complications**			
I	4 (26.7)	4 (28.6)	0.486
II	10 (66.7)	8 (57.1)	
III	0 (0)	1 (7.1)	
IV	0 (0)	1 (7.1)	
V	1 (6.7)	0 (0)	
**Readmissions**	2 (13.3)	3 (21.4)	0.564

*CT* chemotherapy, *ICU* intensive care unit, *SD* standard deviation, *VAS* visual analog scale

## Discussion

This pilot study reported our initial experience in implementing a prehabilitation program for ovarian cancer patients undergoing NACT and interval cytoreductive surgery, being the first study assessing the impact of a prehabilitation intervention. The main results showed a nutritional improvement among patients included in the prehabilitation group that may contribute to reduce perioperative complications and also to improve postoperative recovery.

Prehabilitation programs consist of the patient’s preparation between diagnosis and surgery to improve functional capacity and metabolic reserves before intervention. Previous studies have shown that multimodal prehabilitation programs in major cancer surgeries have a positive impact on the patients’ outcomes [13, 14].

Patients in the prehabilitation group showed higher pre- and postoperative serum total protein and postoperative albumin levels. Given that baseline nutritional parameters at diagnosis were similar between the groups, the combination of both physical exercise and protein supplements 30 min after exercise training could have enhanced muscle hypertrophy and impacted body mass composition and nutritional status. In this line, in patients with rectal cancer having neoadjuvant chemoradiotherapy, prehabilitation was linked to an increase in patients’ muscle mass [20].

Overall, up to 40% of controls showed intraoperative complications vs. 14.3% of patients in the prehabilitation group; however, the potential role of nutritional status on reducing perioperative and postoperative complications is still under investigation. Interestingly, 2 (13.3%) patients in the control group presented anastomosis breakdown, while no patients in the intervention group suffered this postoperative complication. Whether anastomosis breakdown and other postoperative complications could be related to poor nutrition status is a hypothesis that cannot be ruled out. It should be taken into account that complications after surgery are related to surgical parameters but also depend on patient factors [21]. A study of patients with colorectal cancer resection showed no significant beneficial reduction in postoperative complications following nutritional supplementation, although patients who received whey protein supplementation 4 weeks before surgery had a mean improvement in functional walking capacity [22]. Similarly, a prospective randomized study in non-malnourished patients undergoing abdominal cancer surgery showed that patients who received nutritional supplementation for 14 days before surgery significantly reduced postoperative complications. The laboratory parameters decreased in the control group, and in the nutritional supplementation group, they were stable (albumin and total protein) or raised (transferrin and total lymphocyte count) after surgery [23]. Besides, protein supplementation alone has been shown to improve nutritional parameters. Yi et al. randomized 118 patients undergoing elective surgery for gynecological cancer to preoperative carbohydrate-only loading versus whey protein-infused carbohydrate loading. The whey protein-infused carbohydrate loading group had shorter hospital stay, lower readmission rate within 1 month, lower weight loss, lower C-reactive protein/albumin ratio, preserved muscle mass, and better handgrip strength when compared to the preoperative carbohydrate-only loading group [24].

In our study, an improvement in preoperative nutritional status was observed. In this way, several studies in advanced ovarian cancer patients correlated a low serum preoperative albumin and prognostic nutritional index with a worse overall survival [25, 26]. In addition, two prospective studies about major abdominal surgery correlated an early postoperative serum albumin and total protein drop with postoperative complications [27, 28]. Overall, poor preoperative nutritional status reflects poor postoperative nutritional status, which is associated with higher postoperative morbidity. Moreover, a review of Obermair et al. suggests that receiving perioperative nutritional interventions 1–2 weeks prior to surgery and nutritional interventions of early postoperative feeding can reduce the length of hospital stay and postoperative complications in gynecological cancer patients undergoing major surgery [29].

Overall, preoperative carbohydrate loading has been shown to be an effective method to control postoperative insulin resistance [30, 31]. However, data are limited concerning the effects of carbohydrate loading on preoperative hyperglycemia and insulin management in patients with ovarian cancer patients previous to interval cytoreductive surgery. This study adds evidence for recommending routine preoperative carbohydrate loading. Preoperative carbohydrate loading in our prehabilitation group was not associated with intraoperative increased risk of hyperglycemia, postoperative insulin requirement neither intraoperative nor postoperative complications. Similarly, Alimena et al. reported that carbohydrate loading was associated with an increase in preoperative glucose values without impacting the complication rates [31].

Our results also showed that the prehabilitation program was safe with no adverse events or increase of perioperative complications. Although there are few studies available about prehabilitation and postoperative complications, published data suggest a potential benefit of the prehabilitation program in terms of postsurgical readmissions rates and postoperative complications [13, 32]. Systematic reviews and meta-analysis of prehabilitation programs for patients undergoing major abdominal surgery and oncologic surgery reported that prehabilitation programs are feasible and safe with a protective factor for postoperative complications [33–36].

Interestingly, our results showed that the prehabilitation group reported significantly lower requirements of intraoperative blood transfusion rate and a trend towards lower intraoperative vasoactive drug requirement. In this line, a randomized control trial of patients submitted to elective major abdominal surgery also showed a trend toward lower requirements of vasoactive drugs during surgery in the prehabilitation group (*p* = 0.053) [13]. This fact could suggest that prehabilitation would improve tolerability during surgery avoiding hypotension and hypovolemic shock.

Although the recommended length for a prehabilitation period is 2–4 weeks before surgery [15], in the present study, the length of the prehabilitation program was larger, since depended on patients’ disease and individual response to NACT. The period on NACT treatment is a great opportunity to improve functional capacity through a prehabilitation program. Given that type of surgical intervention, surgical complexity, and clinical characteristics were well-balanced between the groups, the duration of the prehabilitation mainly depended on NACT tolerance and toxicity. Interestingly, a shorter time between diagnosis and interval cytoreductive surgery in the prehabilitation group was observed suggesting a better tolerability to chemotherapy in prehabilitated patients. Other studies also demonstrated that prehabilitation program during neoadjuvant treatment is feasible, safe, and well-tolerated in patients with esophagogastric cancer and it has positive effects in increasing exercise capacity before surgery and lower risks of postoperative pneumonia [10, 11, 32, 37].

### Strengths and weaknesses

This study has several strengths that deserve to be commented on. To our knowledge, this is the first study assessing the impact of prehabilitation programs in patients with ovarian cancer undergoing interval cytoreductive surgery after NACT. Moreover, in our center, prehabilitation program is a routine preoperative assessment of gynecologic oncologic patients before surgery. Additional strengths include an homogeneous study population both at demographics and clinical characteristics with advanced-stage ovarian cancer patients undergoing interval cytoreductive surgery after 3 cycles of NACT.

On the other hand, potential weaknesses of our study include the non-randomized control trial design and the inherent limitations associated with a retrospective study, where some data or complications may have been missed. However, the enrollment of all consecutive patients reduced the chance of selection bias, and clinical stage and comorbidities were well matched between the groups. In addition, it was a pilot study with small sample size and, therefore, limited statistical power. As this was a preliminary study, the sample size was decided arbitrarily, and the results may not be able to properly detect the precise differences. Besides, the single-center design may have limited the generalizability of the results. However, a qualitative data collection method was used before surgery to ensure that patients included in the final analysis followed prehabilitation recommendations. Although no formal data analysis of adherence and acceptability was performed due to the clinical setting of the study, the study results may be feasibly imputed to the prehabilitation program.

Finally, it has to be noted that the COVID-19 outbreak may have had an impact on the results, given that organizational aspects of healthcare providers were truncated by this pandemic and the study was carried out in two different scenarios. Besides, the physical and psychological impacts of lockdown on patients included cannot be ruled out.

### Implications for practice and future research

Our findings seem to suggest preoperative and postoperative nutritional improvements in prehabilitated patients, but given the preliminary nature of the design, this study is mainly intended to stimulate further investigations to assess the efficacy of surgical prehabilitation in gynecologic oncology patients, specifically in patients with ovarian cancer. Our findings warrant future prospective evaluation and support the investigation of prehabilitation programs. Moreover, the study provides data contributing to the larger body of evidence and also for eventual reviews or meta-analysis to help clarify the value of prehabilitation programs in ovarian cancer patients.

Future larger and adequately powered studies will help to evaluate the effect of prehabilitation on postoperative surgical, physical, and metabolic outcomes, while investigating tolerance to NACT and oncologic outcomes and periodically evaluate success with quality improvement initiatives. Finally, although standardized prehabilitation guidelines for ovarian cancer patients need to be established, we encourage all groups to prehabilitate ovarian cancer patients undergoing interval cytoreductive surgery, based on the ERAS guidelines.

## Conclusions

In conclusion, a trimodal prehabilitation program comprising exercise, nutritional supplementation, and psychological support administered during NACT may improve the patient’s nutritional status and, therefore, be associated with a better postoperative recovery in patients with advanced-stage ovarian cancer before interval cytoreductive surgery. The period during neoadjuvant treatment is a great opportunity to improve the patient’s functional capacity through a prehabilitation program.

## Data Availability

The datasets generated and/or analyzed during the current study are not publicly available but are available from the corresponding author (Miralpeix, Ester) on reasonable request.

## Abbreviations

SPSS: Statistical Package for Social Sciences
NACT: Neoadjuvant chemotherapy
ERAS: Enhanced Recovery After Surgery
ICU: Intensive care unit
SGS: Surgical complexity score
SD: Standard deviation
